# Genome-Wide Identification of SSR and SNP Markers Based on Whole-Genome Re-Sequencing of a Thailand Wild Sacred Lotus (*Nelumbo nucifera*)

**DOI:** 10.1371/journal.pone.0143765

**Published:** 2015-11-25

**Authors:** Jihong Hu, Songtao Gui, Zhixuan Zhu, Xiaolei Wang, Weidong Ke, Yi Ding

**Affiliations:** 1 State Key Laboratory of Hybrid Rice, College of Life Sciences, Wuhan University, Wuhan, China; 2 Wuhan Vegetable Scientific Research Institute, Wuhan National Field Observation & Research Station for Aquatic Vegetables, Wuhan, 430065, China; Youngstown State University, UNITED STATES

## Abstract

Genomic resources such as single nucleotide polymorphism (SNPs), insertions and deletions (InDels) and SSRs (simple sequence repeats) are essential for crop improvement and better utilization in genetic breeding. However, the resources for the sacred lotus (*Nelumbo nucifera* Gaertn.) are still limited. In the present study, to dissect large-scale genomic molecular marker resources for sacred lotus, we re-sequenced a Thailand sacred lotus cultivar ‘Chiang Mai wild lotus’ and compared with the reported lotus genome ‘Middle lake wild lotus’. A total of 3,180,059 SNPs, 328, 251 InDels and 14,191 SVs were found between the two genomes. The functional impact analyses of these SNPs indicated that they may be involved in metabolic processes, binding, catalytic activity, etc. Mining the genome sequences for SSRs showed that 191,657 SSRs were identified with a frequency of one SSR per 4.23 kb and 103,656 SSR primer pairs were designed. Furthermore, 14, 502 EST-SSRs were also indentified using the available RNA-seq data in the NCBI. A subset of 150 SSRs (genomic and EST-SSRs) was randomly selected for validation and genetic diversity analysis. The genotypes could be easily distinguished using these SSR markers and the ‘Chiang Mai wild lotus’ was obviously differentiated from the other Chinese accessions. This study provides considerable amounts of genomic resources and markers for the quantitative trait locus (QTL) identification and molecular selection of the species, which could have a potential role in various applications in sacred lotus breeding.

## Introduction

Sacred lotus (*Nelumbo nucifera* Gaertn.) is a perennial aquatic plant with high ecological, ornamental and economic value. Due to its edible rhizomes, seeds and leaves, lotus has been cultivated as a vegetable or food for over 7,000 years in Asia. It is also used as an herbal medicine for treatment of cancer, depression, diarrhea, heart problems and insomnia [1, 2]. In addition, its seeds have exceptional longevity, remaining viable for as long as 1,300 years [1]. Although self-pollination is possible, Nelumbo also has cross-pollination, which is usually out mediated by insects. The resultant heterozygosity can be maintained as long as lotus undergoes vegetative propagation via rhizomes [3]. Previous genetic diversity studies have demonstrated that sacred lotus has moderate polymorphism [4, 5]. We have also found that the Chiang Mai wild lotus from Thailand has higher genetic diversity than the Chinese lotus [6, 7]. Carrying a number of beneficial traits, Thai lotus has been particularly useful for developing a series of molecular markers for breeding.

As one of the ancient land plants in angiosperms, the published sequencing data of *N*. *nucifera* genome provided great insights for accession improvement through molecular breeding and unique features, including the longevity of its seeds and adaptation to aquatic environments [8, 9]. The genetic variability of the lotus genomes can be utilized to enhance biotic and abiotic stress tolerance and to improve agronomic traits, such as quality, maturity, and yield potential [10]. Generally speaking, types of variation at the whole-genomic level include microsatellites or simple sequence repeats (SSRs), single nucleotide polymorphisms (SNPs), insertions and deletions (InDels, short insertion and deletion of 1 to 5 bp), and various types of structure variations (SVs). Assignment of molecular markers to linkage groups and construction of genetic map are important for analyzing the genome of species. SSR and SNP markers have gradually become the preferred markers for many applications in genetic and genomic studies, for distributing throughout the genome [11, 12]. Furthermore, as effective and stable markers, SSRs and SNPs play an important role in molecular aided selection and breeding. Although a large number of SSR markers have been developed and 4,098 SNPs have been obtained for the F_1_ population derived from a cross between *N*. *nucifera* ‘China Antique’ and *N*. *lutea* ‘AL1’ using restriction-site associated DNA sequencing (RAD-Seq) technology [13, 14], there are still no sufficient markers for linkage mapping, genome wide association studies (GWAS), QTL analysis, and map-based cloning in scared lotus.

EST-derived SSRs can be related to functional genes, have more evolutionarily conserved characteristics within and across related species and have been widely used for comparative mapping of related crops or genetic diversity of wild and cultivated accessions [15, 16]. Moreover, EST-SSRs may represent the transcripts that contribute to important agronomic traits [17]. Thus, they are useful for molecular marker assisted selection breeding (MAS), with molecular markers either originating from a gene of interest or co-segregating a gene with a desirable agronomic trait. However, very few molecular markers linked to a desirable gene locus have been found in sacred lotus. Recently, only 39 EST-SSR primers and the genic SSR markers that are related to flower buds have been reported [6, 13, 18]. The lack of tightly linked markers for agronomically important genes (such as rhizome development) limit their utilization in the selection of traits of interest in sacred lotus MAS breeding.

With sequencing of the sacred lotus genome, re-sequencing of lotus accession has led to the discovery of millions of SNPs and InDels, which will enable genome-wide association studies (GWAS) to be made for identifying agronomically important genes in Nelumbo [19]. In rice, more than 3.6 million SNPs were found and used in GWAS for 14 agronomic traits through sequencing 517 rice landraces [20]. Currently, available linkage maps in sacred lotus have been constructed using SRAP and RAD-seq, a few SSR markers and recently published SNP-based map [13, 14]. SSR markers have been widely used for constructing linkage maps, quantitative trait locus (QTL) mapping, and MAS for their ubiquity and high level of polymorphism [21]. For instance, using the soybean whole-genome sequences, locus-specific SSR markers were found and 33,065 high-polymorphic SSRs were developed [22]. These results showed that genetic markers such as SSRs and SNPs are abundant in different crop genomes and can be found from the genome sequences, making it more accessible to breeders and geneticists.

Although the *N*. *nucifera* genome has already been sequenced and annotated, the absence of its genomic resources such as SNPs, InDels and SSR markers make it difficult to carry out molecular breeding of *N*. *nucifera*. Furthermore, only 2200 ESTs are currently available in the public NCBI databases. Therefore, in order to accelerate research for this Nelumbo species, there is an urgent need to enrich the available genomic resources. Based on the *de novo* sequencing data of ‘Middle lake wild lotus’, we have re-sequenced the whole genome of ‘Chiang Mai wild lotus’ using the Illumina platform in the present study, and used the available sequencing data to mine for the SSR and SNP markers. These data could be a useful resource for construction of high density genetic maps, high-throughput QTL mapping, improving marker-assisted breeding, and transgenic approaches.

## Materials and Methods

### Plant materials

The samples (*N*.*nucifera* Gaertn.) used in the experiment are maintained by Wuhan National Germplasm Repository for Aquatic Vegetables (30°12′N, 111°20′E), Hubei, People’s Republic of China. Young leaves of Chiang Mai wild lotus were harvested and total genomic DNA was extracted using the cetyltrimethylammonium bromide (CTAB) method [6].

For validation and analysis of genetic diversity, a total of 24 *N*. *nucifera* accessions were taken for the present study (complete details are given in S1 Table). Total genomic DNA was extracted from fresh young leaves using the modified CTAB method as previously described [6]. DNA quality and quantity were determined by agarose gel electrophoresis and Nanodrop2000 (Thermo) spectrophotometry.

### Library construction, genome re-sequencing and assembly

DNA libraries were constructed with an insert size of 500bp and sequenced using the high throughput Illumina Hiseq2000 to produce 2×100 paired-end reads on the Illumina Hiseq2000 platform. The published genome sequences of ‘Middle lake wild louts’ were used as a reference genome in this study [9]. We mapped all the reads to the pseudomolecule of the reference genome through SOAP2 and then sorted these by the coordinates [23]. The obtained mapping results were used to detect variations. The raw sequence data obtained have been deposited at the NCBI in the Short Read Archive (SRA) database under the accession number: SRP061673.

### Detection of SNPs and InDels among cultivars of sacred lotus

‘Chiang Mai wild lotus’ and ‘Middle lake wild lotus’ are two cultivars from different regions, which can be differentiated using the SNPs and InDels. To ensure the SNPs and InDels between the two cultivars were not due to misassembled contigs, we mapped the raw data of ‘Chiang Mai wild lotus’ to the *N*. *nucifera* pseudomolecule sequences using the Burrows-Wheeler Alignment (BWA) algorithm. Then using SOAPsnp and SOAPindel, the SNPs and InDels (1 to 5 bp) between the two cultivars were identified, respectively [24].

SNPs were filtered by the quality value given by SOAPsnp, which should be >20, and the base quality at this position should pass the rank-sum test (in SOAPsnp with P >0.05). Unique SNPs showing ≥ 10 read depths were considered as reliable SNPs. The reliable SNPs were further confirmed by double-checking the raw assembly data with alignment view to reduce false positives. The non-synonymous changes in CDS regions were chosen for further analysis and GO analysis and enrichment were performed by WEGO and ArgiGO, respectively. Each SNP and InDel was annotated by SnpEff (http://snpeff.sourceforge.net/index.html) to predict the effects of variants on genes.

Structural variation (SVs) is another important variation among different individuals of the same species. Detection and annotation of the variation can help us to understand and explain the difference of different individuals. The input files included the mapping result of each accession, the gap information of the reference genome, and the insert-size of the mapped paired-end reads. According to the mapping results, a remarked difference between the gap information and the insert-size of paired-end reads usually indicates candidate SVs, including deletions, duplications, and inversions. SOAPsv was used to identify SVs in this study.

### SNPs validation using PCR and Sanger sequencing

To validate the accuracy of SNPs prediction between the cultivars ‘Chiang Mai wild lotus’ and ‘Middle lake wild lotus’, 32 randomly chosen SNPs which induce amino acid changes in the coding sequence (CDS) region, were selected for validation using PCR and Sanger sequencing. The two cultivars ‘Chiang Mai wild lotus’ and ‘Middle lake wild lotus’ were used for verifying the SNP sites. Primer pairs were designed to amplify the flanking sequence of selected SNPs using Primer 3 (http://bioinfo.ut.ee/primer3-0.4.0/). All primers are shown in S2 Table. PCR was performed in 25μL reaction volumes using the following conditions: denaturation 95°C for 3min, 40 cycles of amplification (95°C for 30s, 56°C for 40s, and 72°C for 1 min), and a final extension of 72°C for 10 min. The amplified PCR products were purified, cloned and sequenced and then analyzed by BioEdit v7.0.5.3 (http://www.mbio.ncsu.edu/BioEdit/bioedit.html).

### SSR identification, validation and diversity analysis

The genomic sequences of ‘Chiang Mai wild lotus’ obtained from resembled resequencing data and RNA-seq data were used for the SSR motif search, respectively [8]. EST contigs were generated for RNA-seq data from GenBank Short Read Archive raw data (Accession SRX266474, SRX266489, SRX268456 and SRX265003) using the *de novo* assembly method (Trinity) [25]. A non-redundant dataset of unigene sequences was then created using paired-end reads, which ensures the distance between different contigs from the same transcriptome. The program MISA (MIcroSAtellite identification tool) (http://pgrc.ipk-gatersleben.de/misa) was used to identify localize microsatellite motifs in the *N*. *nucifera* genome and EST contigs. Only perfect SSRs, including mono-, di-, tri-, tetra-, penta-, and hexa-nucleotide motifs with numbers of uninterrupted repeat units more than 10, 7, 6, 5, 4 and 4, respectively, were targeted. The SSR loci that are used for developing genetic markers should include a perfect repeat motif and two unique flanking sequences with 200 bp on each sides of the repeat [15]. The sequences containing EST-SSRs were searched for functional domain markers (FDM) using InterProScan (http://www.ebi.ac.uk/Tools/InterProScan/) [26].

The forward and reverse primers were designed based on unique flanking sequences using Batch Primer 3 (http://primer3.sourceforge.net/). The SSR loci were only considered to contain two to six nucleotides motifs with a minimum of 6, 5, 4, 4 and 4 repeats, respectively. Mononucleotide repeats were excluded. The parameters for designing PCR primers were as follows: (1) primer length ranging from 18 to 22 bases with optimal sizes of 20nt; (2) PCR product size range of 100 to 300 bp; (3) melting temperature between 55°C and 63°C, with 60°C as the optimum annealing temperature; (4) a GC content of 40%-60%, with an optimum of 50%.

To validate the genomic SSR (gSSR) and EST-SSR markers, 80 and 20 primer pairs were chosen for PCR amplification, respectively (S2 Table). PCRs were performed in a 15μL volume containing 25 ng of genomic DNA. The PCR reactions were carried out in a MyCycler^™^ Thermal Cycler (Bio-RAD) using the following conditions: initial denaturation at 95°C for 3 min, 35 cycles at an annealing temperature ranging from 56 to 60°C for 30 s, 72°C for 1 min, and a final extension at 72°C for 7 min. The PCR products were separated on 6% denaturing polyacrylamide gel, and the genotype was scored after silver staining. The number of alleles was recorded and allelic data of all the genotypes were analyzed by POPGENE version 1.32 [27]. The polymorphism information content (PIC) was calculated as described by Anderson (1993): PIC = 1-Σ*P2ij*,where *Pij* is the frequency of the *j*th allele for *i*th locus [28]. The Jaccard’s similarity coefficient was used to estimate pair-wise similarity coefficients between pairs of genotypes. Based on the similarity matrix, dendrograms were constructed using the unweighted pair group method with arithmetic mean (UPGMA) clustering method. The reliability and robustness of the dendrograms were tested using bootstrap analysis with 1,000 replicates [29]. The above analyses were performed using modules in NTSYS-PC software (version 2.2) [30].

## Results

### Sequences assembly and variations detection

Raw Illumina sequencing read data were filtered out with a custom perl script to trim the low-quality or adapter sequences of both ends. Sequencing errors in the illumine data were corrected by String Graph Assembler (SGA) software v 0.0.20 with k-mer -55 [31]. We mapped paired-end reads to the reference genome using BWA 0.7.6a with the default parameters. Only uniquely mapped and paired aligned reads were used for detecting variations [32].

The genome size of ‘Chiang Mai wild lotus’ is approximately 811, 218, 286 bp, slightly larger than that of the reference genome (‘Middle lake wild lotus’, 792, 334, 941bp). Pseudomolecules of the ‘Chiang Mai wild lotus’ were constructed from 24, 986.28Mb sequences. Compared with the reference genome, a total of 3, 180, 059 SNPs, 328, 251 InDels and 14,191 SVs were detected (Table 1 and Table 2). Among these, most of the SNPs were observed at the intergenic region with a frequency of one per 2.18 kb of the reference genome. Only 2.93% were located in coding sequence (CDS) regions (S3 Table). Furthermore, 23,436 synonymous and 36,323 non-synonymous SNPs were identified (Table 3 and S4 Table). The ratio of non-synonymous to synonymous substitutions was 1.55, which is higher than that of rice (1.29) [33], but lower than that of soybean (1.61) [34]. Most of the InDels were homozygous, and insertion or deletion of SVs accounted for 92.05% of the all SVs (Table 3).

**Table 1:** Statistics of genomic resources developed from assembled data of ‘Chiang Mai wild lotus’.

Resources developed	
Total no. of raw reads (M)	347.93
Total no. of clean reads (M)	277.62 (79.79%)
Raw data (Mb)	31,314.42
Clean data (Mb)	24,986.28
GC content	40.30%
Total of SNPs with Middle lake lotus	3,180, 059
Total of indels with Middle lake lotus	328,251
Total of SVs with Middle lake lotus	14,191

**Table 2:** Statistics of single-nucleotide polymorphisms (SNPs), insertions and deletions (InDels) and SVs detected between ‘Chiang Mai wild lotus’ and ‘Middle lake wild lotus’.

	Chiang Mai wild lotus-Middle lake wild lotus		
	SNPs	InDels	SVs
Chr01	187 990	21 476	865
Chr02	215 274	23 256	977
Chr03	207 465	22 661	948
Chr04	215 756	22 256	1014
Chr05	212 440	20 914	860
Chr06	232 684	22 407	985
Chr07	218 487	23 002	878
Chr08	209 330	22 627	932
Chr09	207 486	22 006	947
Chr10	195 005	20 063	842
Chr11	200 190	21 048	924
Chr12	206 890	22 698	974
Chr13	204 009	20 925	909
Chr14	200 077	20 171	824
Chr15	189 462	18 721	879
Chr16	77 514	4 020	433
**Total**	**3 180 059**	**328 251**	**14 191**

**Table 3:** Statistics of variations between ‘Chiang Mai wild lotus’ and reference genome ‘Middle lake wild lotus’.

**SNP**	**CDS -Non_syn**	**CDS -Syn**	**Intron**	**Intergenic**	**Splice_intron**
	36,323	23,436	213,550	1,765,495	236
**Indel**	**Insert**			**Delete**	
	**Heterozygous**	**Homozygous**		**Heterozygous**	**Homozygous**
	36,399(21.85%)	130,165(78.14%)		20,546(12.71%)	141,141(87.29%)
**SV**	**Insertion**	**Deletion**		**Duplication**	**Others**
	5,438(38.32%)	7,625(53.73%)		1,055(7.43%)	73(0.51%)

CDS: coding site; Non_syn: non-synonymous mutations; Syn: synonymous mutations.

### Distribution and functional analysis of SNPs

**Fig 1:**
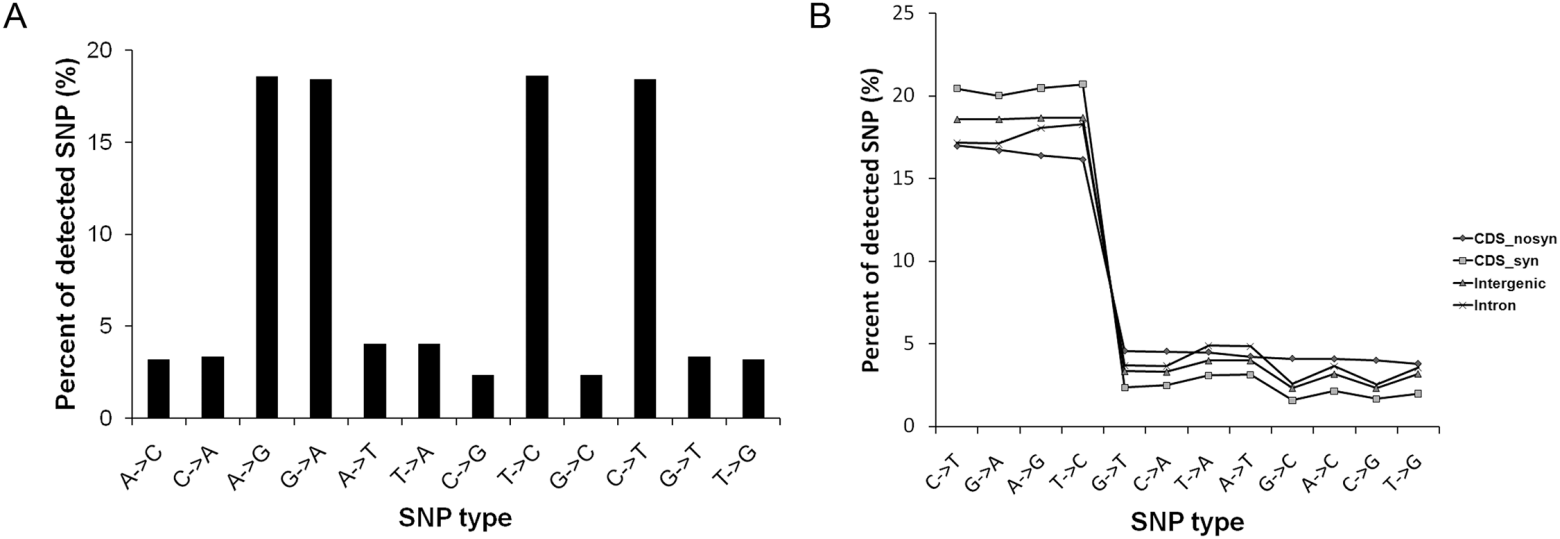
Distributions of different single-nucleotide polymorphism (SNP) types. (A) The frequency of different SNP types; (B) different SNPtypes in coding sequence (CDS)-nosyn, CDS-syn, intergenic and intron regions.

**Fig 2:**
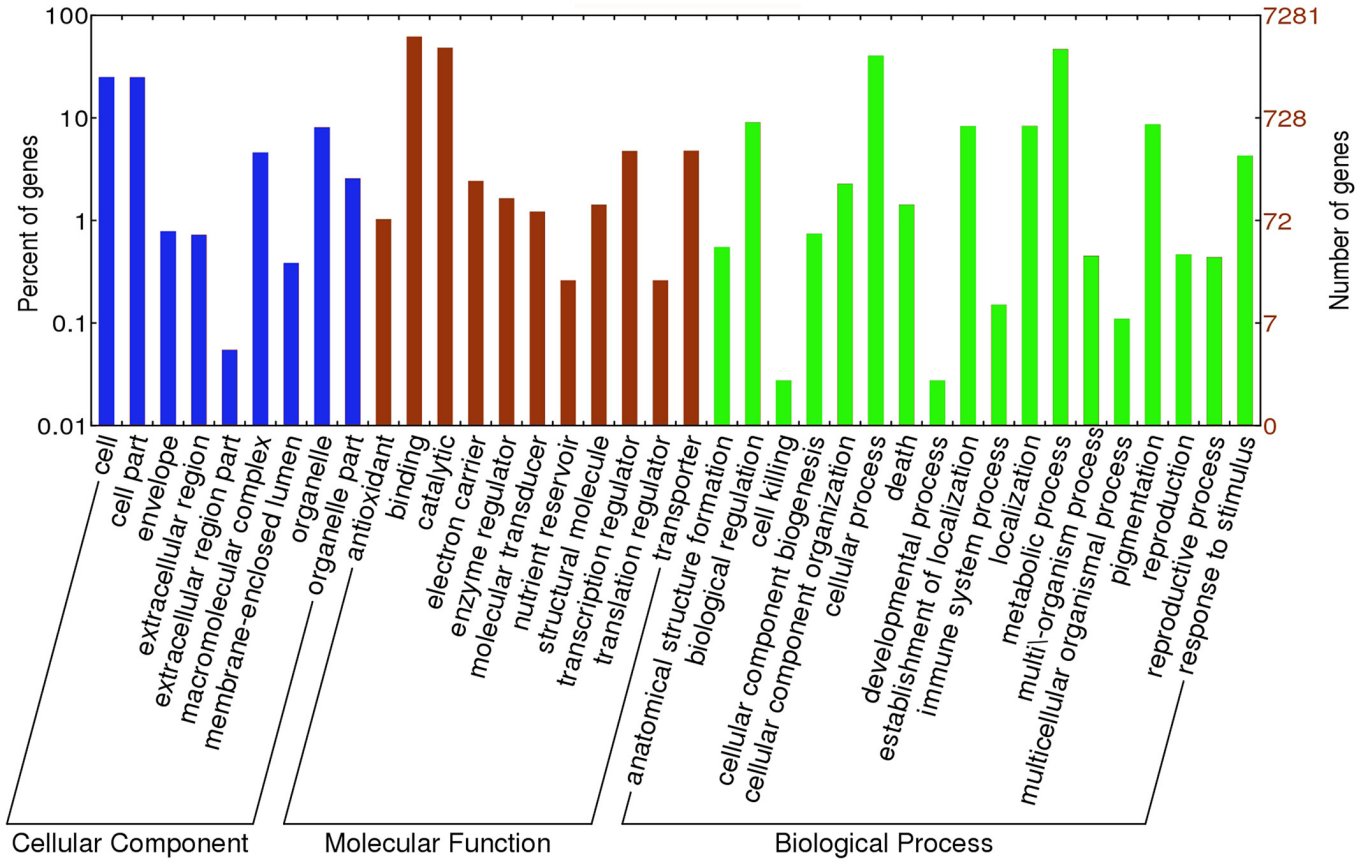
Functional annotation of the genes with non-synonymous single-nucleotide polymorphism (SNP) in coding sequence (CDS) into different gene ontology (GO) categories (biological process, molecular function and cellular components).

Distribution analysis of SNPs showed that A/G and C/T transitions accounted for 36.99% and 37.04%, respectively, and G/C, G/T, A/C and A/T transversions accounted for 4.71%~8.12% of all SNP types (Fig 1A). The non-synonymous transition of A/G and C/T transitions were more abundant in CDS regions. However, many other SNP types were synonymous and were also found in CDS regions (Fig 1B). The percentage of base substitutions was comparable to that found in previous studies [34, 35]. To further annotate the function of the non-synonymous SNPs in coding genes, GO analysis was conducted for three categories: Cell Component (CC), Molecular Function (MF) and Biological Process (BP). The results showed that they were involved in many processes (Fig 2 and S5 Table). The most abundant components of the CC categories are “cell (GO:0005623)” and “cell part (GO:0044464)”. In the MF category, the most abundant component are “binding (GO: 0005488)”, followed by “catalytic activity (GO:0003824)”. As for the BP terms, a great number of the genes are assigned to “cellular process (GO:0009987)”, “metabolic process (GO:0008152)” and “pigmentation (GO: GO:0043473)”. GO enrichment of the non-synonymous SNPs in coding genes also showed that “metabolic process”, “binding” and “catalytic activity” were the abundant terms (S6 Table).

### Validation of SNP events

To investigate the validation rate of the SNPs identified by bioinformatic analysis in this study, 32 SNPs were verified by PCR amplification and Sanger sequencing. Of these, primer pairs of 27 SNPs can amplify target sequences. After sequencing comparison, 22 SNPs were validated in ‘Chiang Mai wild lotus’ and ‘Middle lake wild lotus’. The estimated prediction accuracy reached 81.5% (Table 4).

**Table 4:** Summary of single-nucleotide polymorphism (SNP) validation in ‘Chiang Mai wild lotus’ and ‘Middle lake wild lotus’.

Location of fragment	Gene ID	Annotation	Number of SNPs tested	Number of SNPs validated
**NELegrR_Vchr1:59907..60530**	CUFF.31519.1		3	3
			60007 C<->A	60007 C<->A
			60428 A<->G	60428 A<->G
			60430 T<->A	60430 T<->A
**NELegrR_Vchr2:31416721..31417669**	NNU_21394-RA	Similar to FBXW11: F-box/WD	2	
		repeat-containing protein 11	31367445 G<->A	-
			31367446 G<->A	-
**NELegrR_Vchr5:18375031..18375494**	NNU_05281-RA	Similar to RBM28: RNA-binding protein 28	4	4
			18375131 T<->C	18375131 T<->C
			18375146 C<->G	18375146 C<->G
			18375272 A<->G	18375272 A<->G
			18375394 G<->A	18375394 G<->A
**NELegrR_Vchr6:13264307..13264779**	NNU_05281-RA	Similar to MYB3R-1: Myb-related protein 3R-1	4	4
			13264407 C<->A	13264407 C<->A
			13264650 C<->T	13264650 C<->T
			13264663 G<->A	13264663 G<->A
			13264679 G<->C	13264679 G<->C
**NELegrR_Vchr7:21698807..21699438**	NNU_09299-RA	Similar to LIP1: Lipoyl synthase 2C	3	3
		mitochondrial	21698907 A<->C	21698907 A<->C
			21698958 G<->C	21698958 G<->C
			21699076 C<->T	21699076 C<->T
**NELegrR_Vchr9:18773204..18773771**	NNU_15030-RA	Similar to XTH21: Probable xyloglucan	3	1
		endotrans glucosylase/hydrolase protein 21	18773304 A<->G	18773304 A<->G
			18773474 T<->C	-
			18773671 G<->A	-
**NELegrR_Vchr14:17854380..17854740**	NNU_25833-RA	Similar to GH22778: Protein KIAA0664 homolog	2	2
			17854480 G<->A	17854480 G<->A
			17854640 T<->C	17854640 T<->C
**NELegrR_Vchr16:7441441..7442066**	NNU_16559-RA	Similar to sraP: Serine-rich adhesin for platelets	6	5
			7441541 A<->G	7441541 A<->G
			7441600 A<->G	7441600 A<->G
			7441640 C<->A	-
			7441651 C<->T	7441651 C<->T
			7441795 T<->G	7441795 T<->G
			7441966 C<->T	7441966 C<->T

### Mining of genomic SSRs and EST-SSRs

In the present study, all the assembled contig sequences were used to search microsatellites using MISA software with a criterion of a minimum 5 repeat motifs for each SSR type. A total of 191,657 SSRs were identified with the frequency of one SSR per 4.32 kb in the genome. The sequences flanking the SSRs were used to design primers, and a total of 103, 656 SSRs were designed (Table 5, S7 Table, and S8 Table). The most abundant types of repeat motif were di-nucleotide repeats (27.08%) followed by tri-nucleotide repeats (11.58%). The frequencies of SSRs based on number of motifs revealed that SSRs with 5~15 tandem repeat motifs were the most common (S4 Table). Of the di-nucleotide motifs, AG/CT were the most frequent (20.65%), followed by (AT)n (3.67%). Of the tri-nucleotide motifs, AAG/CTT were the most abundant (5.40%) followed by AAT/ATT (2.10%) (S7 Table and S8 Table).

**Table 5:** Classification of simple sequence repeats (SSRs) and expressed sequence tag-SSRs (EST-SSRs) repeats.

**Genomic-SSR data**	
Total size of sequences (bp)	811, 217, 991
Number of SSRs	191, 657
Number of compound SSRs	12, 137
Frequency of SSR in genome	1/4.23kbp
**EST-SSR data**	
Number of seqs searched	52, 717
Total size of sequences (bp)	54, 424, 069
Number of SSRs	14, 502
Number of SSR contining seqs	10, 619
Number of seqs containing more than one SSR	2, 815
Number of compound SSRs	1, 229
Frequency of SSR in transcript	1/3.75kbp

To date, few EST-SSR markers have been found in sacred lotus [6]. In this study, based on the RNA-seq data of four different tissues, 14,502 repeat motifs were found in sacred lotus (Table 5). Most of the repeat types were dinucleotides and the dominant classes of sequence repeat were AG/CT (33.83%) (S9 Table). After removing the SSRs located at the ends of sequences, 3,432 primer pairs were designed (S10 Table).

The Functional Domain Markers (FDM) were found from the EST-SSRs containing sequences using InterProScan [26]. Totally, 2278 SSR containing sequences were analyzed and 798 SSR-FDMs were identified (S11 Table). The functional domains were responsible for Protein kinase domain, Pyridoxal phosphate-dependent transferase, Small GTP-binding protein domain,FAD dependent oxidoreductase, PDZ-binding protein, RNA recognition motif, etc (S11 Table).

**Fig 3:**
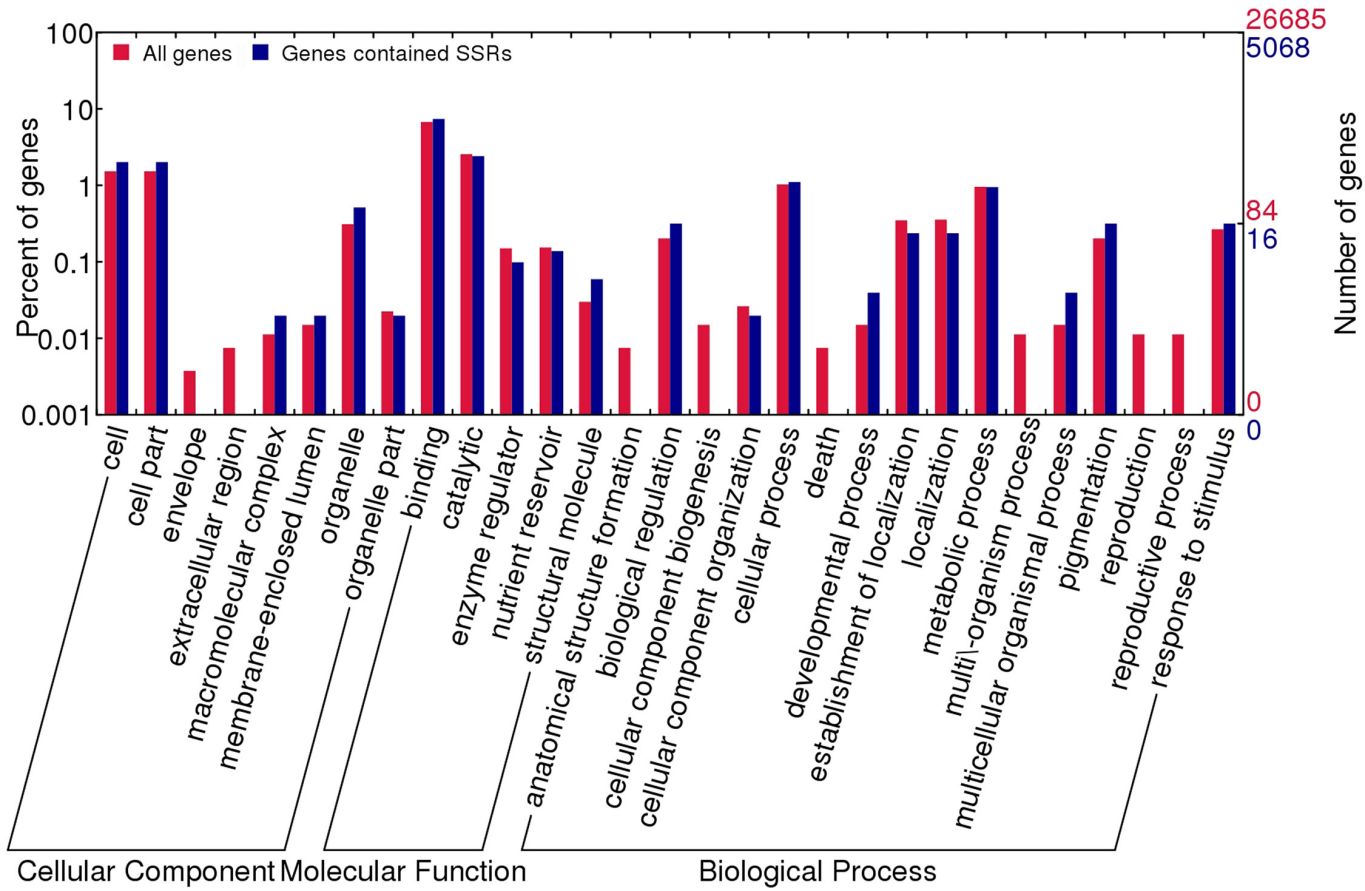
Functional annotation of the genes with simple sequence repeats (SSRs) into different gene ontology (GO) categories (biological process, molecular function and cellular components).

GO annotation was performed for the transcripts containing SSRs using all sacred lotus genes as the background (Fig 3). With regard to biological processes, genes involved in the “developmental process”, “multicellular organismal process” and “response to stimulus” were highly represented. In terms of molecular function, “structural molecule” was the most abundant GO term. Regarding cellular components, the major categories were “cell”, “cell part” and “macromolecular complex” (Fig 3).

### Validation of SSRs and analysis of genetic diversity

To validate the SSRs, 120 primer pairs of the genomic SSR markers were randomly selected for PCR amplification. Forty primer pairs of them produced clear bands and 35 polymorphic primer pairs were further used for the analysis of genetic diversity within 24 sacred lotus accessions (S1 Table). To evaluate the reliability of the EST-SSR primers, 30 primer pairs were randomly selected for PCR amplification and 7 primer pairs showed successful amplification (Table 6).

**Table 6:** Characteristics of the polymorphism of 35 genomic simple sequence repeat (SSR) and 7 expressed sequence tag (EST)-SSR primers in 24 sacred lotus accessions.

loci	alleles	Repeat motif	Forward primer	Reverse primer	PIC	Size (bp)
**NnSSR05**	5	(AAT)22	ATGGCTGAATGACATGTTGG	TCCATTGACACCCCTACCAT	0.65	120–163
**NnSSR08**	4	(CATA)17	TGCCCTAGCCTAGCCCTTAC	TGGGGTTCAGGTTTGTCAAT	0.61	155–215
**NnSSR09**	4	(TACA)14	TGAGGCTCGCAAGCATAGTA	CGAGCTCGACTATAAGCCTTT	0.45	285–303
**NnSSR11**	6	(ATAC)22	ATCCCCCTCCCTTCTCTCTA	ACAAGAGGGAGAAGAATTACGA	0.75	175–250
**NnSSR13**	7	(ATGT)15	CTTAGATTTTCCCGCGCATC	TGATGCCTTGCGATTTGATA	0.81	195–250
**NnSSR15**	2	(TTC)21	GAAAACTGGTCCTTGAAAGTGC	CACATCCTGACACATGAGAGC	0.04	200–250
**NnSSR17**	9	(TAA)27	CGGGTGGTGATTTCATTGTT	GGTCTTCCTCAAAACTCTCACG	0.83	160–220
**NnSSR21**	6	(ATA)23	GGGGATTACCGTTAGGCTGT	CAGTCCAACGTTCAATTGGTT	0.74	175–200
**NnSSR23**	5	(TA)37	TGGGTTGATTTTCAGTTTGC	TCGACTGACCCAATCACAAA	0.66	255–280
**NnSSR27**	5	(ATT)25	AACGGAGCCTTAATCCCATT	TCTTCATCGGCATTCAATCA	0.53	270–305
**NnSSR28**	6	(TAT)20	CCAAGTTGAATTGTCGAACC	AAAGGTGAGCATTGTTGTTG	0.70	160–215
**NnSSR29**	5	(TAT)24	CCGGCCACGTGCTTAGTAT	AGGATCAACAAGATGGAGAAGG	0.64	225–260
**NnSSR30**	10	(TAT)18	TCCCAAGATTACCCCAACTTT	TGAGGGACTTGATAAGATGCAG	0.83	145–225
**NnSSR36**	4	(ACAATA)16	TGCACTGCTGTTACATGAGAAA	GCCTCATGCACACCTCATAA	0.61	270–385
**NnSSR41**	7	(TAT)43	AAAACAATGGCCCCATACAT	TTCCTCCCATGTAACTTGAACT	0.60	140–250
**NnSSR42**	3	(AT)55	GTTCCCCATGGGACTCAAAT	CCCAGACTCCTTACCCAATG	0.42	450–520
**NnSSR45**	7	(AAT)38	GTCGCGGGTACTTGAGAAAT	CCTTGCCGACCTGTGTTATT	0.68	90–160
**NnSSR46**	6	(TTC)30	TTGGCTCTCACCTCTCACAA	GGCTCTCACAAGTGGATCGT	0.68	110–155
**NnSSR47**	5	(TAA)38	CCTACTGCAATTCCCTCCTG	TTAAAAATCAGCCGCACCAT	0.73	200–270
**NnSSR48**	5	(TCT)34	CTGCAACCTGCAAGTCCTTC	TGAGAAAATTGTCGGCTGAA	0.52	265–290
**NnSSR49**	8	(ATT)34	GATGATTGGACGGACACTCC	GGAAGTGCGGAACAGACAAT	0.78	160–225
**NnSSR50**	4	(TTA)31	AAGTTGGAGCTCGATTTCAGA	TCATGAGCCGGTTCAAATAA	0.58	270–295
**NnSSR51**	5	(ATA)33	CGTCACGGGTACCTACGAAA	GCTCTCCCTGCTGACCTGTA	0.60	105–138
**NnSSR55**	6	(TAT)53	CGATGTGCTCTCTCCTTTTG	GGCAGAGACCTCTCGGTACA	0.72	170–210
**NnSSR56**	5	(TTA)43	TTGCTCCCCTTATTGACCTG	TCTCGGTTTCTTCCCCACTA	0.62	95–115
**NnSSR58**	8	(ATT)33	TAAAAGGGCCTACCCTGTCG	CGTCATAAGGCGACCGTAAC	0.71	95–170
**NnSSR59**	8	(GA)30	TTTGCATTGACAACGAGAGC	GACATGCTCGGTGACTCGTA	0.76	130–183
**NnSSR60**	4	(CT)31	TCTCCGACGGTGACCAAGT	CGATGCCTGAGTTCGTCTCT	0.56	165–185
**NnSSR62**	8	(AGA)28	AATTCGAGGAGGAGGAGGAG	TGCTGGTAAAGTTGTGGGAAG	0.73	120–205
**NnSSR63**	5	(TCT)25	TCGACCCATTTTTCAAAAGC	GGCAGGGGAGGAAATGTTAG	0.61	140–165
**NnSSR64**	6	(ATT)28	CCGAAAATCCGTCTAGAATCA	TCATCGGGTCGGTTTAGGTA	0.75	195–310
**NnSSR66**	5	(AGAT)12	CCAGAAGGGTTTCTTCGAGTT	TTTCAGGTGTACCCAAACGTC	0.68	185–215
**NnSSR67**	5	(AAG)16	CCGCTCTGGTCATTTCTAGC	CCCACTTCCAATCTCCCTCT	0.70	200–235
**NnSSR68**	9	(AAT)27	CCTCTGGCCCTATCGAGAAT	AGTGGCCAGTGCCACATATC	0.80	195–280
**NnSSR69**	4	(GAA)28	GTTCGCGGTTTGAGAAATTG	CGGTAACACAGTGCAGACGA	0.59	155–175
**EST-SSR03**	3	(GAG)12	TCAGATCCCATCACGAAGGT	CAACCCGACACGAAGAAATC	0.50	141–152
**EST-SSR06**	2	(GA)13	AGTCGGTGCCTTCACCATT	CCACTGCAAACAAGACAAGG	0.37	142–152
**EST-SSR09**	2	(GA)12	TGAGTGGAGTTGGGTTTTCA	TCGTTAACACCACTTGTTTGTG	0.38	102–110
**EST-SSR15**	4	(TGC)7	AGAAAGTGGCTGCATTGCTT	GCATTGATTCAGCAGCAGAG	0.56	150–166
**EST-SSR21**	2	(GCT)7	CATCCTCCTCCACTGTTTCC	AATTGCTACCAACCCGCTTT	0.37	230–235
**EST-SSR26**	2	(GGC)8	AATCGTCGAAGAAGCAGACC	CTCCTTCGCCGTCGTTATTA	0.37	150–159
**EST-SSR30**	2	(TAT)8	TTTACAACGCTGTGCACTCA	GACCGCAAGGACATGCTTAT	0.35	260–266

**Fig 4:**
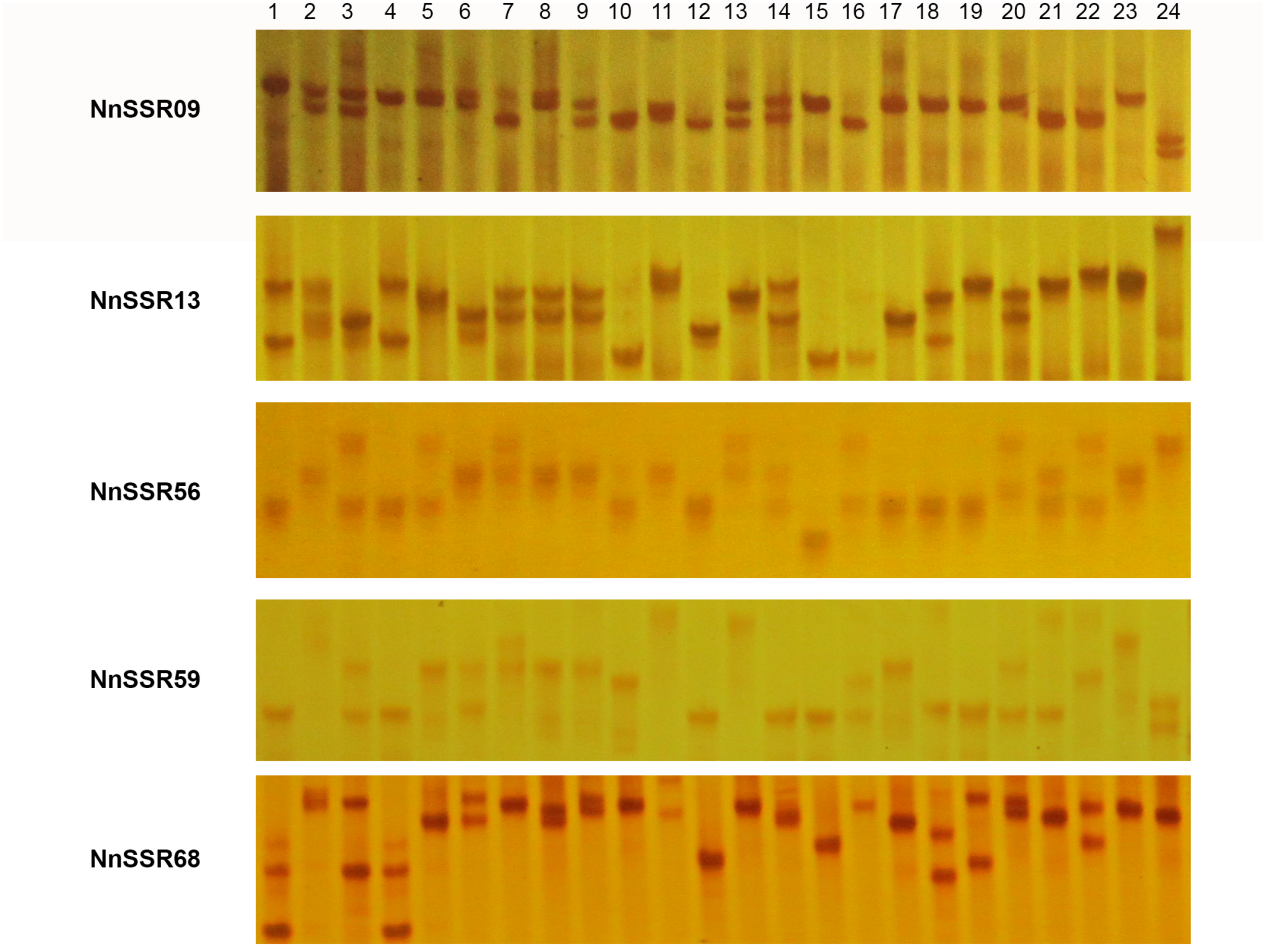
Alleic polymorphism among 24 accessions of *Nelumbo nucifera* by microsatellite marker NnSSR09, NnSSR13, NnSSR56, NnSSR59, NnSSR68. Lanes 01–24 (genotypes see S1 Table); M, 20bp Ladder (500,400, 300, 200,180,160,140bp).

For genomic SSRs, the number of alleles per locus ranged from 2 to 10, with an average of 5.74 alleles per locus (Fig 4, Table 6 and S12 Table). The polymorphic information content (PIC) for these markers ranged from 0.04 to 0.83 with an average of 0.65. Among the 35 loci, the observed and expected heterozygosity (*H*_O_ and *H*_E_) ranged from 0.000 to 0.583 (mean 0.291) and from 0.042 to 0.867 (mean 0.707), respectively (S12 Table). These results are consistent with those reported previously [19]. The number of alleles per locus and the observed and expected heterozygosity (*H*_O_ and *H*_E_) of EST-SSRs ranged from 2 to 4, 0.1429 to 0.952, and 0.467 to 0.645, respectively (S12 Table). And the PIC of these EST-SSR markers ranged from 0.35 to 0.56 with an average of 0.41, which was lower than that of genomic SSRs (Table 6 and S12 Table).

**Fig 5:**
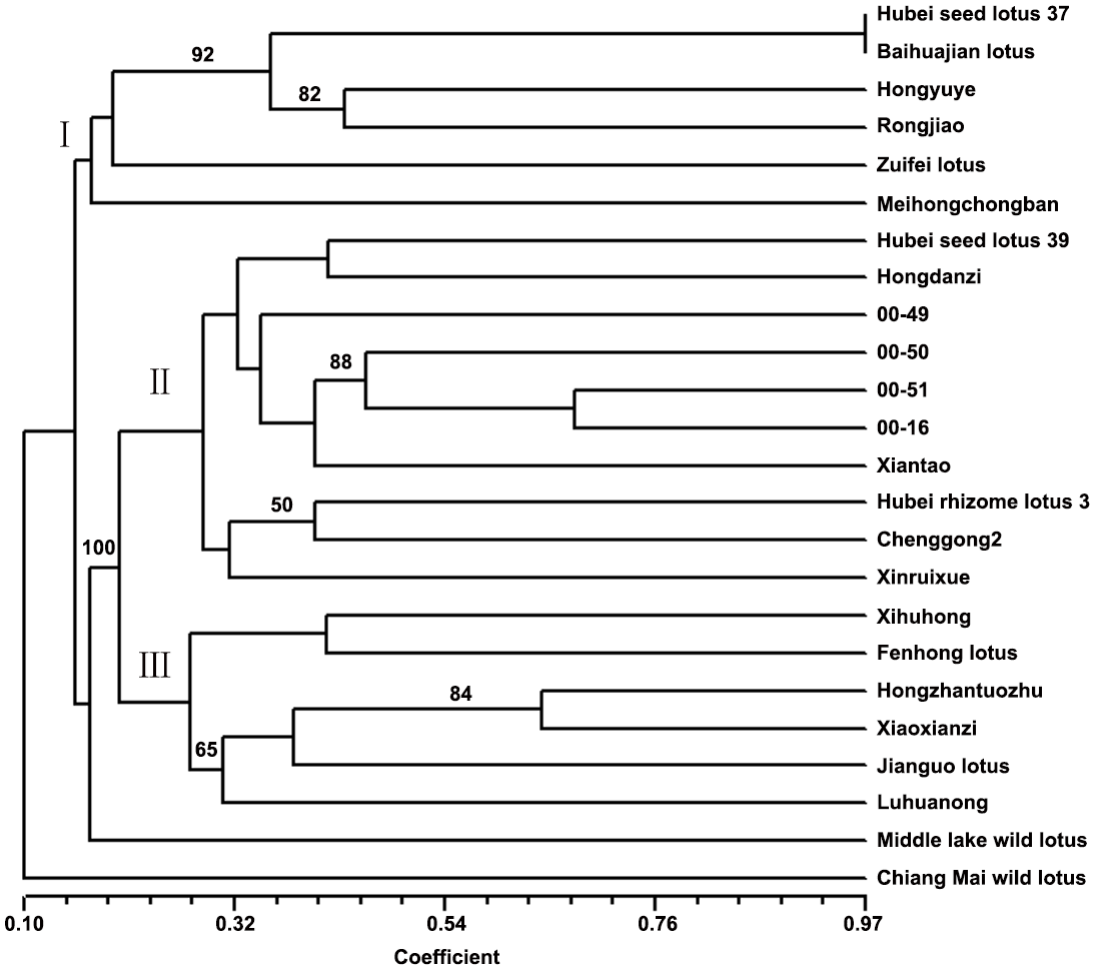
Dendrogram generated using unweighted pair group method with arithmetic mean (UPGMA) for 24 accessions based on 35 genomic simple sequence repeat (SSR) and 7 expressed sequence tag-SSR (EST-SSR) markers.

The 42 polymorphic SSR loci produced a total of 220 alleles across all the genotypes. Further genetic relationships among the 24 accessions were determined from an unweighted pair-group method of arithmetic averages (UPGMA)-based dendrogram (Fig 5). The genetic similarity coefficient between genotypes based Jaccard’s method, varied from 0.10 to 0.97. In addition to the two wild lotuses, other cultivated lotuses can divided into three groups and the group III was flower lotuses (Fig 5 and S1 Table). Group I included two seed lotuses (‘Hubei seed lotus 37’ and ‘Baihuajian lotus’) and flower lotuses. The most complicate was group II, containing seed lotuses, rhizome lotuses and flower lotuses. However, the three lotuses were generally clustered together, except for the flower lotus (‘Xiantao’) and rhizome lotus (‘Hubei rhizome lotus 3’). Therefore, most of the accessions were distinguished by the SSR markers. In particular, the Thai lotus was distinctly differentiated from the Chinese lotuses. And the wild lotus accessions (‘Middle lake wild lotus’ and ‘Chiang Mai wild lotus’) were also differentiated (Fig 5).

## Discussion

Previous studies which used ISSR, AFLP and SSR markers have shown that the lotus accession in Thailand was genetically as well as morphologically different from the Chinese lotus [6, 7]. In the present study, we re-sequenced the genome of the ‘Chiang Mai wild lotus’ and detected variations (SNPs, InDels and SVs) with that of ‘Middle lake wild lotus’ in China (Tables 2 and 3). The results of the statistics of SNPs and InDels detected between the two accessions showed that there are more than 3 million SNPs, three-hundred thousand InDels, and ten thousand SVs (Table 2). These variations will provide useful genomic resources for future studies of genetic differentiation.

Because of the abundance, SNP/InDel molecular marker is a useful alternative to SSR in high density marker studies, such as quantitative trait locus (QTL) identification, genetic map construction and fine genetic mapping [35]. With the rapid development of next generation sequencing, genome-wide SNPs/InDel was much easier to discovery. In this study, a total of 3,180,059 SNPs, and 328,251 InDels were identified in the *N*. *nucifera* genome, which was much higher than that detected from the transcripts [36].

Microsatellites or SSRs are distributed widely and randomly in eukaryotic genomes. Further, although SNPs serve as excellent markers for high-throughput mapping and studying complex genetic traits, SSRs have several advantages for their co-dominant, hyper-variability, polymorphism, ease and reliability of scoring [21, 37]. As a useful genetic marker, SSR has been used extensively for analysis of genetic diversity, population genetics, linkage mapping and association analysis [38]. Furthermore, the high PIC value of SSRs (up to three fold higher than SNPs), coupled with high heterozygosity values makes them useful for assessment of genetic relatedness and map base cloning [39]. Because of the unavailability of SNPs and few SSR markers in the sacred lotus, the SSR identification in the present study offers a resource for the geneticists and breeders.

In this study, RNA-seq data from four tissues (leaf, petiole, root and rhizome internode) were used for developing 3,432 EST-SSR markers. Although the polymorphic of EST-SSRs is less than that of genomic SSRs in this study, they can also be used for genetic diversity analysis of the sacred lotus (Fig 5). Moreover, EST-SSRs are easily transferable across species, more advantageous for revealing adaptive differentiations at the population level. And they are distributed in coding sequences and may be related to functional genes [40]. Because EST-SSRs were developed from four different tissues, they may co-segregate with some functional genes and could be used as a potential tool for MAS breeding. This will further facilitate gene cloning and functional studies of genes involved in lotus rhizome internode growth and development.

The analyses of genetic diversity among the sacred lotus genotypes clearly established for fairly high PIC values of genomic SSR markers. And even closely related sacred lotus genotypes could be distinguished. The average number of alleles per locus observed in our study (5.74) was higher than that in previous studies of the sacred lotus (3.8 and 3.33) [7, 37], but comparable to that of the American Nelumbo (5.77) [4]. This difference could be due to a number of SSR markers developed in our study and high PIC SSRs could be easily to be chosen. Moreover, the motif repeats of the polymorphic SSRs were higher than that of previous studies. The difference between the average observed heterozygosity (0.291) and expected heterozygosity (0.707) may suggest the occurrence of self-pollination within the population (S10 Table).

In this study, the dendrogram showed that the wild lotuses were clearly separated from the cultivated lotuses, especially, the ‘Chiang Mai wild lotus’ was distinctly differentiated from the Chinese lotuses (Fig 5). The results were consistent with those of previous studies, indicating that the wild lotus and other lotus cultivars may have experienced different divergence patterns [5, 7]. Most of the flower lotuses were differentiated from seed lotuses and rhizome lotuses, while some of them could be clustered. This may be because seed lotus, flower lotus and rhizome lotus are classified by their good agronomic characters of beautiful flower, high yielding seeds or high-quality rhizomes in the process of domestication. However, these different lotus types (seed lotus, flower lotus or rhizome lotus) may have similar genetic background.

In summary, our study contributes a considerable amount of genomic resources for the sacred lotus, including SNPs, genomic SSRs and EST-SSRs. Utilization of this genomic information in linkage mapping, comparative genomics and molecular breeding will need considerable efforts, which would facilitate improvement of the sacred lotus.

## Conclusions

In the present study, we generated more than 2.5 million DNA sequences by resequencing the ‘Chiang Mai wild lotus’ genome. Compared to the reference genome ‘Middle lake wild lotus’, a total of 3,180,059 SNPs, 328, 251 InDels and 14,191 SVs were detected. Using the DNA sequences and available RNA-seq data in the NCBI, we identified 191, 657 genomic SSRs and 14, 502 EST-SSRs for the sacred lotus. A total of 150 SSR primer pairs (120 genomic-SSR and 30 EST-SSR primer pairs) were designed in this study, of which 42 SSR were validated for amplification and showed polymorphism. Using these primers, genetic diversity across 24 accessions of *N*. *nucifera* was examined and distinguished. We believe that these SNPs and SSRs will be valuable genetic resources for constructing linkage maps, quantitative trait locus (QTL) mapping, genetic diversity and MAS breeding in *N*. *nucifera*.

## Supporting Information

S1 Table List of the 24 genotypes used for the analysis of genetic diversity. (XLSX)

S2 Table All the primers of single-nucleotide polymorphism (SNP) and simple sequence repeat (SSR) makers used in this study. (XLSX)

S3 Table All the single-nucleotide polymorphism (SNP) in the coding sequence (CDS) region between the ‘Chiang Mai wild lotus’ and ‘Middle lake wild lotus’ genomes. (XLSX)

S4 Table All the non-synonymous single-nucleotide polymorphism (SNP) substitutions in the coding sequence (CDS) region between the ‘Chiang Mai wild lotus’ and ‘Middle lake wild lotus’ genomes. (XLSX)

S5 Table Gene ontology (GO) analysis of the non-synonymous single-nucleotide polymorphism (SNP) substitutions in the coding sequence (CDS) region between ‘Chiang Mai wild lotus’ and ‘Middle lake wild lotus’ genome. (XLSX)

S6 Table GO enrichment of the non-synonymous SNPs in the coding sequence (CDS) region between ‘Chiang Mai wild lotus’ and ‘Middle lake wild lotus’ genome. (XLSX)

S7 Table Frequency of classified repeat types of genomic simple sequence repeats (SSRs). (XLSX)

S8 Table Detailed information of genomic simple sequence repeat (SSR) loci of the sacred lotus (*Nelumbo nucifera*) identified in the study. (XLSX)

S9 Table Frequency of classified repeat types of expressed sequence tag-simple sequence repeats (ESR-SSRs). (XLSX)

S10 Table Detailed information of expressed sequence tag-simple sequence repeat (EST-SSR) loci of the sacred lotus (*Nelumbo nucifera*) identified in the study. (XLSX)

S11 Table Identification of the simple sequence repeat- Functional Domain Markers (SSR-FDMs) in sacred lotus (*Nelumbo nucifera*) (XLSX)

S12 Table The polymorphisms of 35 simple sequence repeats (SSRs) and 7 expressed sequence tag-SSRs (EST-SSRs) in sacred lotus accessions. All information about the polymorphic primers, number of alleles (*N*), observed heterozygosity (*H*_o_), expected heterozygosity (*H*_e_), and polymorphism information content (PIC) is shown.
(XLSX)
